# The Effect of Revascularization on Lower Limb Circulation Parameters in Symptomatic Peripheral Arterial Disease

**DOI:** 10.3390/jcm13133991

**Published:** 2024-07-08

**Authors:** Andreas L. H. Gerken, Martin Sigl, Elisa Israel, Christel Weiß, Christoph Reißfelder, Kay Schwenke

**Affiliations:** 1 Department of Surgery, University Medical Center Mannheim, Medical Faculty Mannheim, Heidelberg University, Theodor-Kutzer-Ufer 1-3, D-68167 Mannheim, Germanychristoph.reissfelder@umm.de (C.R.); kay.schwenke@umm.de (K.S.); 2 Department of Cardiology, Angiology, Haemostaseology and Medical Intensive Care, University Medical Centre Mannheim, Medical Faculty Mannheim, Heidelberg University, Theodor-Kutzer-Ufer 1-3, D-68167 Mannheim, Germany; martin.sigl@umm.de; 3 Department of Medical Statistics and Biomathematics, University Medical Center Mannheim, Medical Faculty Mannheim, Heidelberg University, Theodor-Kutzer-Ufer 1-3, D-68167 Mannheim, Germany; christel.weiss@medma.uni-heidelberg.de

**Keywords:** peripheral arterial disease, microcirculation, lightguide spectrometry, oxygen to see

## Abstract

**Background:** The prevalence of peripheral arterial disease and the number of revascularization procedures performed in symptomatic patients are steadily increasing. However, uncertainties remain regarding hemodynamic monitoring after revascularization and the prediction of clinical outcomes. This study aimed to investigate hemodynamic parameters with a focus on the microvasculature. **Methods:** This prospective, single-center study included 29 patients (15 with intermittent claudication [IC] and 14 with chronic limb-threatening ischemia [CLTI]). Before and after the revascularization procedure, in addition to the ankle–brachial index (ABI), microperfusion parameters, including microvascular blood flow, capillary oxygen saturation (SO_2_), and relative hemoglobin content (rHb), were assessed with lightguide spectrophotometry combined with laser Doppler flowmetry using an oxygen-to-see (O2C) device in the horizontal and elevated leg positions. **Results:** At baseline, SO_2_ in the elevated leg position was significantly lower in patients with CLTI than in those with IC (*p* = 0.0189), whereas the other microcirculatory parameters and ABI values were not significantly different. Patients with diabetes mellitus had a higher flow rate than those without in the horizontal leg position (*p* = 0.0162) but not in the elevated leg position. After successful revascularization, the flow increased immediately and significantly in both positions, whereas SO_2_, rHb, and the ABI did not. **Conclusions:** Elevated leg SO_2_ was significantly lower in CLTI than in clinically compensated peripheral arterial disease, whereas microvascular flow was a suitable surrogate parameter indicating successful revascularization. In studies using surgical or interventional revascularization procedures, noninvasive hemodynamic monitoring of the microcirculation at the foot level might be beneficial.

## 1. Introduction

The worldwide prevalence of peripheral arterial disease (PAD) and the number of revascularization procedures performed on the lower extremities are steadily increasing [1,2]. Restoring arterial perfusion through endovascular or open surgical interventions aims to improve quality of life and limb preservation and reduce the morbidity and mortality associated with PAD. In patients with intermittent claudication (IC), the focus is on extending their walking distance; in the stage of chronic limb-threatening ischemia (CLTI), the aim is to prevent amputations and their consequences [3].

Despite the therapeutic progress in PAD treatment, particularly in endovascular techniques, its clinical success still requires improvement. A systematic review showed that 1 year after endovascular or surgical revascularization, 40% of wounds in patients with diabetic foot syndrome and PAD persist, and 1 in 10 patients even undergo a major amputation [4].

Hence, the importance of the microvasculature for the function and maintenance of the lower extremities is increasingly being emphasized, not least because of advances in medical technology in terms of the qualitative and quantitative recording of microcirculation. Impaired microcirculation is associated with a 4-fold increased risk of amputation, and a combination of PAD and microvascular disease is associated with a more than 20-fold risk [5]. Microcirculation not only plays a role in CLTI or diabetic foot syndrome but also in the clinical stage of IC in patients without diabetes mellitus [6].

Currently, revascularization procedures are mainly accompanied by diagnostic assessment of the macrovasculature, whereas microvascular changes are rarely monitored peri-procedurally [7]. This study aimed to investigate hemodynamic parameters, focusing on the microcirculation, before and after revascularization procedures.

## 2. Materials and Methods

### 2.1. Study Design, Patient Selection, and Clinical Assessment

In this single-center prospective pilot trial, patients with symptomatic PAD scheduled for lower limb revascularization were enrolled at the Interdisciplinary Vascular Center of the University Medical Center Mannheim, Germany, between February 2020 and June 2021. The eligibility criteria included Rutherford categories 2–5. The patients with IC (Rutherford 2–3) showed no or marginal improvement after a 3–6 month period of conservative treatment, including medical therapy, lifestyle changes, and exercise training. Patients with congestive right heart failure, myocardial infarction, erysipelas, or wounds and major tissue loss (Rutherford 6) located in the area of the measurement points were excluded. We aimed to enroll consecutive patients; however, logistical problems and special precautions during the pandemic disrupted the recruitment. Clinical assessments included demographic data, the clinical stage of PAD according to the revised Rutherford classification, cardiovascular comorbidities, and risk factors. In addition, current medications were recorded.

### 2.2. Circulation Parameters and Data Acquisition

#### 2.2.1. Macrocirculation

Lower limb PAD imaging included invasive angiography during the revascularization procedure and assessment of the ankle–brachial index (ABI) before the procedure and on the first postoperative day. Measurement was performed using a Doppler probe, 8–10 MHz, placed in the area of the pulse at a 45–60° angle to the skin. The ABI values were calculated by dividing the highest ankle pressure in each leg by the highest arm pressure. Duplex ultrasound was performed to confirm the patency of the treated artery or bypass.

#### 2.2.2. Microcirculation

For microcirculation assessment, we used an oxygen-to-see device (O2C), version III (LEA Medizintechnik GmbH, Giessen, Germany), the physical principles of which have been described in detail previously [8]. Briefly, the microlight-guided O2C device combines white light spectrometry (wavelength range, 500–630 nm) and laser Doppler flowmetry (wavelength, 830 nm). This combination permits the measurement of post-capillary hemoglobin oxygen saturation (SO_2_, in %) and relative microvascular hemoglobin (rHb, in arbitrary units [AUs]) using spectrometry. Simultaneously, the Doppler shift in the laser light can be used to assess the microvascular blood velocity (in AU). The relative microvascular blood flow (in AU) was calculated using rHb and velocity. Using an LFX-29 probe (LEA Medizintechnik GmbH, Giessen, Germany) with a measurement duration of 10 s, the average values of the measurement periods were used for the subsequent analyses.

The measurements were carried out before revascularization, immediately after revascularization, and during the postoperative hospital stay, at the earliest 1 day after revascularization. All the measurements were performed according to a standardized protocol after a resting period of a minimum of 10 min in the supine position at three plantar foot locations (medial forefoot, lateral forefoot, and heel) using double-sided adhesive tape (LTDT-001; LEA Medizintechnik GmbH, Giessen, Germany) in two leg positions (horizontal and elevated leg afterwards, using a 30 mmHg positioning aid). The measurement results for the three measurement sites were averaged to represent the entire foot in one value.

### 2.3. Follow-Up

The study focused on short-term changes in the hemodynamic parameters after revascularization. In addition, we offered patients an optional follow-up examination to record the macro- and microcirculation and determine the functional and clinical outcomes after one to two months. Clinically, we used the wound condition and the occurrence of a reintervention or major amputation as secondary outcome parameters.

### 2.4. Statistics

Prior to our analysis, we performed a sample size calculation. As primary outcomes, we considered differences in the parameters of microperfusion “pre OP–post OP”. Assuming a medium effect (Cohen’s D = 0.6), a sample size of 24 was estimated in order to show that these differences were statistically significant using a paired *t*-test (alpha = 0.05, power = 0.8, 2-sided test). Assuming furthermore a drop rate of up to 20%, 30 patients were required in order to be able to detect changes over time. The sample size calculation was performed with the SAS Procedure PROC POWER. Different statistical tests were performed to evaluate the possible influence of the revascularization procedure on lower extremity microperfusion. A paired *t*-test was applied to compare paired data (e.g., measured values in the horizontal versus elevated leg positions). In order to compare the mean values of the two groups, a 2-sample *t*-test was performed. Multiway analysis of variance (ANOVA) for repeated measurement was performed to simultaneously analyze several factors for each individual microcirculatory parameter, namely SO_2_, rHb, and flow. This statistical method was performed using the SAS procedure PROC MIXED, with the patients’ IDs as a random factor and the time, position, and group as fixed factors. The Scheffé test was performed as a post hoc test. All the statistical calculations were carried out using SAS version 9.4 (SAS Institute, Cary, NC, USA). The results of the statistical tests were considered significant for test results with *p* < 0.05.

## 3. Results

### 3.1. Clinical Characteristics

A total of 29 patients (with IC, *n* = 15; with CLTI, *n* = 14) with symptomatic PAD who underwent revascularization were included in the study, after 1 of the 30 patients recruited withdrew his consent without giving specific reasons. The endovascular therapy included prolonged plain old balloon angioplasty, followed by stent implantation as a bailout option in case of suboptimal angiographic results. No drug-coated balloons, drug-eluting stents, or endovascular debulking techniques were applied. The baseline demographic and clinical characteristics are summarized in Table 1.

### 3.2. Baseline Circulation Values

**Figure 1:**
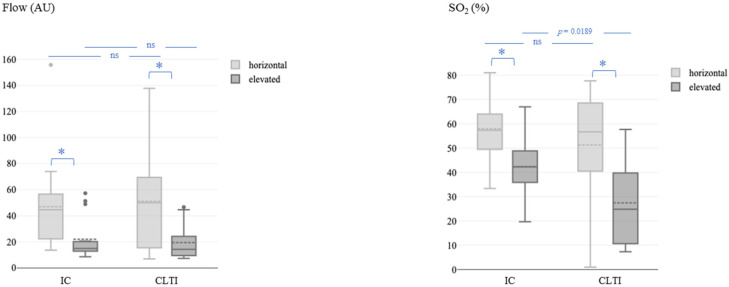
Microvascular blood flow (**left**) and oxygen saturation (**right**) at baseline (before the revascularization procedure) in the horizontal and elevated leg positions. Flow, microvascular blood flow; AU, arbitrary units; SO_2_, capillary oxygen saturation; IC, intermittent claudication; CLTI, chronic limb-threatening ischemia. The asterisk (*) represents a *p*-value < 0.05.

At all the measurement times, microvascular blood flow and SO_2_ were significantly lower in the elevated leg position than in the horizontal leg position in both patients with IC and CLTI, whereas rHb showed no significant differences. At baseline, SO_2_ in the elevated leg position was significantly lower in patients with CLTI than in patients with IC (27% ± 18% vs. 42% ± 13%; *p* = 0.0189), whereas no significant difference was detected in the horizontal leg position, and flow did not differ by CLTI versus IC in either position (Figure 1).

Notably, patients with diabetes mellitus had higher microvascular flow values than those without diabetes when measured in the horizontal leg position (77.60 ± 46.23 vs. 33.93 ± 21.60 AU; *t*-test followed by Satterthwaite post hoc analysis, *p* = 0.0162) but not in the elevated leg position.

### 3.3. Effect of Revascularization

**Figure 2:**
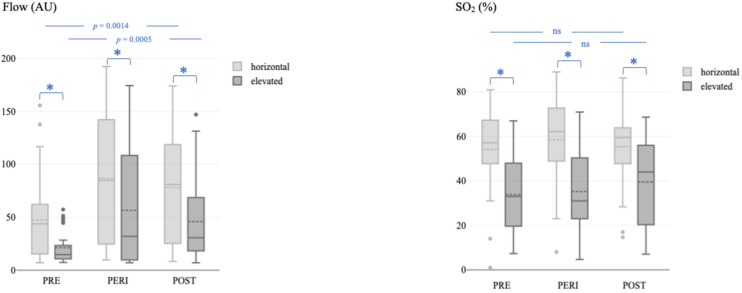
Microvascular blood flow (**left**) and oxygen saturation (**right**) before, immediately after, and during the postoperative course after the revascularization procedure in the horizontal and elevated leg positions. Flow, microvascular blood flow; AU, arbitrary units; SO_2_, capillary oxygen saturation; PRE, before the revascularization procedure; PERI, immediately after the revascularization procedure; POST, postoperative course after the revascularization procedure. The asterisk (*) represents a *p*-value < 0.05.

Multiway ANOVA showed a significant difference between the time of measurement and microvascular blood flow in the horizontal (*p* = 0.0014) and elevated (*p* = 0.0005) positions (Figure 2, left). The Scheffé post hoc comparison test revealed significantly higher flow values immediately after the operation and on the first postoperative day in both positions (all *p* < 0.05). In contrast, the differences between the time of measurement and SO_2_ were not significant, neither in the horizontal (*p* = 0.5375) nor in the elevated (*p* = 0.5022) position (Figure 2, right).

**Figure 3:**
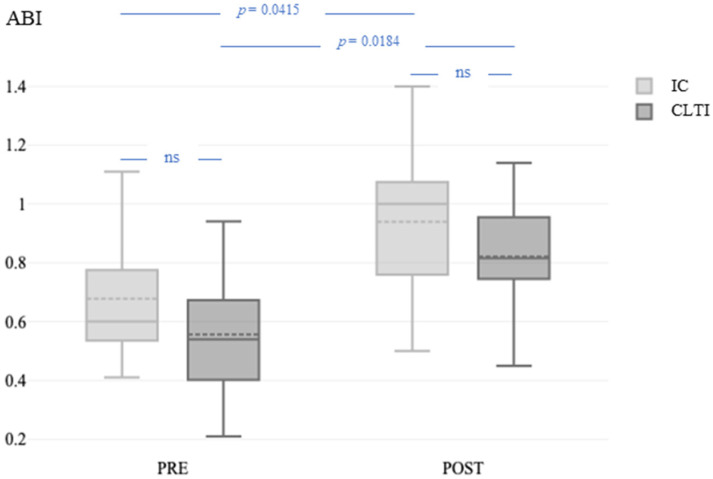
ABI before and 1 day after the revascularization procedure in patients with IC and CLTI. ABI, ankle–brachial index; PRE, before the revascularization procedure; POST, 1 day after the revascularization procedure; IC, intermittent claudication; CLTI, chronic limb-threatening ischemia.

Excluding high ABI values (>1.4) in four patients with incompressible crural arteries, the ABI was not different between patients with CLTI and those with IC at baseline (0.55 ± 0.24 vs. 0.66 ± 0.20; *p* = 0.2314) or 1 day after the revascularization procedure (0.74 ± 0.26 vs. 0.93 ± 0.26; *p* = 0.0947). The ABI was significantly higher after revascularization than at the baseline (Figure 3) in both patients with IC (*p* = 0.0415) and those with CLTI (*p* = 0.0184).

### 3.4. Follow-Up

During the follow-up, a subset of 18 out of the 29 study participants (10 with IC and 8 with CLTI) who underwent revascularization was re-examined 28 to 59 days after revascularization. There were three surgical revisions and no endovascular revisions within the follow-up period. One patient had a bypass thrombectomy due to unilateral occlusion of an aortofemoral bifurcation graft. In two patients with CLTI, a major amputation had to be performed due to uncontrolled infection. Otherwise, the patients with CLTI showed (granulating) wound healing.

The mean values of the O2C parameters are provided as Supplementary Materials (Table S1). ANOVA with repeated SO_2_ measures in the horizontal position showed that there was no significant difference between the variables (*p* = 0.698). In contrast, in the elevated position, we found a significant difference between the variables (*p* = 0.005). A pairwise group comparison revealed statistically relevant differences between preoperative SO_2_ and SO_2_ as measured during the follow-up. Neither microvascular flow nor the ABI values were significantly different compared to the previous measurements.

## 4. Discussion

Symptomatic PAD is associated with microvascular alterations. However, the microcirculation is not routinely examined as part of PAD treatment, even when macroperfusion is specifically addressed during revascularization procedures. In this study, we evaluated the skin perfusion parameters before and after successful revascularization in patients with symptomatic PAD. The main findings of this prospective study are as follows:

1. At baseline, SO_2_ in the elevated leg position was significantly lower in patients with CLTI than in those with IC (*p* = 0.019), whereas the ABI values were not significantly different.
2. Patients with diabetes mellitus had higher flow than those without in the horizontal leg position (*p* = 0.016) but not in the elevated leg position.
3. After successful revascularization, the flow immediately and significantly increased in both positions, whereas SO_2_, rHb, and ABI did not.

The limitations of the ABI as a standard diagnostic tool for PAD are widely known, which include variations in performance; interobserver variability, with the consequence of decreased reproducibility; and a wide range of reported sensitivities and specificities [9].

Various noninvasive tissue perfusion measurements have been used in PAD studies to monitor the effects of revascularization. In a systematic review, Wermelink et al. concluded that the diagnostic accuracy of 10 different techniques was low [7]. The current guidelines provide standard values for a critical perfusion status according to transcutaneous partial pressure of oxygen (TcpO_2_) measurement to estimate the risk and level of amputation. In contrast, the variation in values limits its application as a quantitative measure in patients with IC—i.e., in most of our study patients. In IC patients, the transcutaneous oxygen exercise profile would be a suitable method for investigating peripheral arterial insufficiency during exercise and, in combination with O2C, would be an interesting approach for further studies with PAD subgroups. However, TcpO_2_ assessment is time-consuming, the values depend on many variables, and the data are deficient [3]. The use of an O2C system, as in our study, offers several advantages. It is noninvasive and painless, the measurement is rapid, and it can simultaneously record several microcirculation parameters.

### 4.1. Microcirculation during Rest and under Provocation

In the horizontal leg position, the median value of SO_2_ in our IC group (57%) was identical to that of a previous study. In patients with IC, Gyldenløve et al. [10] determined a median SO_2_ of 57% (depth, 8 mm) in the symptomatic area, measured at the first toe, whereas we obtained the mean value of three plantar positions. In patients with CLTI, Rother et al. found a mean SO_2_ of 46% at baseline [11], which is close to the mean SO_2_ we obtained (51%). In contrast, the mean SO_2_ was 80% in a healthy population, and the values were not significantly influenced by leg elevation [12]. Overall, SO_2_, which primarily records venous capillary oxygen saturation and can be regarded as a surrogate for oxygen extraction, does not appear to serve as a reliable parameter for distinguishing between different clinical stages of PAD. A recent study suggested that oxygen extraction (increasing deoxyhemoglobin levels without significant changes in oxyhemoglobin levels) potentially increases as a function of deteriorating blood flow [13].

Often, measurements under provocation conditions reveal an underlying tissue perfusion deficit. In principle, various provocation maneuvers can be performed, such as leg elevation, treadmill testing (as in the study by Gyldenløve et al. [10]), or local heating [14], which resulted in a significant decrease in SO_2_ in each of these studies. In our study, the mean SO_2_ values in the elevated leg position were significantly lower in patients with CLTI (27%) than in patients with IC (42%), with an extensive range and overlap between the groups. Traditionally, values > 10% indicate amputation level viability in critical limb ischemia [15]. The same threshold value was established for coronary bypass surgery to detect regions of myocardial hypoxia [16].

Importantly, provocation maneuvers also alter microvascular blood flow. The flow was significantly reduced after leg elevation in our study and upon treadmill testing in the limbs in the study by Gyldenløve et al. [10]. In the present study, the mean flow in patients with PAD before revascularization was 47 AU at rest, which decreased to 22 AU under elevation, as opposed to that of a healthy population, whose mean flow was 77 AU measured in the horizontal leg position and 66 AU while elevated [12]. In contrast to SO_2_, we are unaware of any validated cutoff values for microvascular flow indicating critical ischemia or amputation risk. However, a single value seems insufficient to assess perfusion status, and a set of microvascular parameters is needed for comprehensive characterization of tissue supply.

### 4.2. Controversy of Microcirculation in Diabetes Mellitus

We found a higher microvascular blood flow in patients with diabetes mellitus in the horizontal leg position than in those without diabetes. In contrast, no significant difference was observed in the elevated leg position. Definitely, the fundamental difference between PAD and diabetic foot syndrome is the presence of neuropathy in the latter [17]. However, there has been ongoing controversy regarding microvascular disorders in both the early stages of diabetes mellitus and late neuroischemic diabetic foot skin [18]. Our finding of an increased blood flow in patients with diabetes mellitus is congruent with older studies in which increased capillary pressure was measured [19] and supports the historical hemodynamic hypothesis. Hemodynamic changes cause vascular adaptation, including capillary remodeling [20]. It should be noted that a distinction must be made between blood flow through dermal arteriovenous shunts and nutritive blood flow, both of which are thought to be controlled by neurohumoral mechanisms [21]. In diabetic foot syndrome, an increased microvascular flow is not equivalent to a better skin supply. Again, the conditions under provocation may be more meaningful: the microvascular perfusion reserve, assessed using a thallium-201 muscle perfusion scan, in the lower limbs of patients with long-term type 2 diabetes without PAD was significantly reduced compared with that in healthy controls [22]. Similarly, optical coherence tomography-derived measurements of flow revealed slightly higher baseline values in diabetic feet but significantly impaired local heating responses [14].

### 4.3. Microvascular Changes Following Revascularization

After successful revascularization, the microvascular blood flow increased immediately and significantly. In a systematic review, Normahani et al. identified three studies that measured changes in microperfusion after percutaneous angioplasty and bypass operations using laser Doppler, similar to our study. These studies consistently observed improvements in perfusion after revascularization [23]. Our results confirm the findings of Rother et al., who measured microcirculatory changes using an O2C device during tibial angioplasty in 30 patients with CLTI, two-thirds of whom had a diagnosis of diabetes mellitus [11]. We agree with the authors that changes in tissue perfusion are not restricted to angiosome-defined borders. There were significant differences in the ABI increases and SO_2_ in the cited study, in contrast to our results. This is most likely due to the heterogeneity of patient cohorts with partially incompressible arteries (which were excluded from the ABI analysis) and an increase in microcirculation parameters in most, but not all, individuals, as previously reported [24].

While the microvascular flow improved immediately after successful revascularization, the SO_2_ in the elevated position was significantly improved at follow-up compared to the preoperative status. Although the limited number of patients and parameters assessed did not allow for further correlation analyses, it seems plausible to us that adaptive mechanisms are involved. While a number of mechanisms may play a role after reperfusion [25], these could also be disrupted by factors like ischemia–reperfusion injury [26]. This, together with different patient cohorts, revascularization procedures, and measurement methods, could explain the heterogeneity of the results during follow-up. For example, Ma et al. [13] found no significant changes in the TcpO_2_ values compared to the baseline six weeks after endovascular therapy, while the values in another study [27] in patients with IC or CLTI increased significantly during this period. Furthermore, Kaczmarczyk et al. could not detect a significant impact of PTA on indices of endothelial function such as arterial pulse waveform analysis (aPWA), flow-mediated dilatation (FMD), or the reactive hyperemia index (RHI) during a one-year follow-up period [28].

For patients without revascularization options, the effectiveness of and evidence for therapeutic alternatives for improving microcirculation and wound healing are considered low [29]. Recently, a pilot study suggested a positive effect of acupuncture on microcirculation [30].

### 4.4. Limitations

Limited conclusions were drawn because of the small number of patients included in this study. The follow-up in the current study was restricted to 4–8 weeks because the focus was on short-term changes in hemodynamic parameters. Further studies with larger sample sizes and long-term follow-up should be performed to confirm the findings of this study and increase the quality of the data. Furthermore, the characteristics of patients with diabetes mellitus should be examined in more detail. Clarifying the differences between type 1 and type 2 diabetes, the need for insulin treatment, and further complications of diabetes could explain the causal relationships between microcirculation and PAD/diabetes. As O2C spectrometry enables the non-invasive assessment of four dimensions of microcirculation, we deliberately chose not to perform an invasive method, such as TcpO_2_, as a method of reference. This comparison should be the focus of future trials. We are aware that the toe–brachial index may be more sensitive for detecting PAD, especially in the presence of medial calcification, when the ABI is elevated. However, only two patients had ABI values over 1.3 in our cohort.

## 5. Conclusions

Although elevated leg SO_2_ was significantly lower in CLTI than in clinically compensated PAD, microvascular flow seems to be a suitable surrogate parameter indicating successful revascularization in both groups. In studies using surgical or interventional revascularization procedures, noninvasive hemodynamic monitoring of the microcirculation at the foot level might be beneficial; however, larger studies assessing possible confounders are warranted.

## Figures and Tables

**Table 1:** Baseline characteristics of the patients with symptomatic peripheral arterial disease (*n* = 29). Quantitative variables are presented as means ± standard deviation. For qualitative factors, absolute frequencies and percentages (in parentheses) are given.

Age (Years)	68 ± 9
Male	21 (72)
Peripheral arterial disease level	
Aortoiliac	7 (24)
Femoro-popliteal	13 (45)
Cruropedal	9 (31)
Crural run-off *	
One-vessel	6 (29)
Two-vessel	6 (29)
Three-vessel	9 (42)
Rutherford clinical category	
2–3 (IC)	15 (52)
4–5 (CLTI)	14 (48)
Prior amputation (ipsilateral + contralateral)	4 (14)
Type of revascularization	
Bypass	19 (66)
Aorto(bi)iliac	6
Aorto(bi)femoral	4
Iliac-popliteal	1
Femoro-popliteal	3
Femoro-crural/pedal	5
Endarteriectomy	5 (17)
Endovascular (PTA/stent)	3 (10)
Endarteriectomy + endovascular	2 (7)
Target limb ankle–brachial index ^a^	
ADP	0.53 ± 0.3
ATP	0.52 ± 0.3
Cardiovascular comorbidities and risk factors	
Cerebral vascular disease ^b^	8 (28)
Coronary artery disease ^c^	19 (66)
Congestive heart failure (stages)	
Preserved	10 (34)
Mid-range	6 (21)
Reduced	3 (10)
Tobacco consumption	
Previous smokers	8 (28)
Current smokers	18 (62)
Diabetes mellitus	10 (35)
Arterial hypertension	25 (86)
Dyslipidemia	25 (86)
Chronic inflammatory disorders ^d^	0 (0)
Other comorbidities	
Peripheral neuropathy	5 (17)
Preoperative anemia ^e^	14 (48)

Data presented as means ± standard deviation or absolute numbers and proportions (%). * Missing data in eight patients with aortoiliac/aortofemoral revascularization. ^a^ Excluding high ankle–brachial index values (>1.3) in two patients with incompressible crural arteries and ankle–brachial index of 0 in two patients with IC. ^b^ History of transient ischemic attacks or stroke or present (stenosis ≥ 50% in ultrasound) or previously revascularized carotid stenosis. ^c^ Invasively diagnosed coronary artery disease. ^d^ Defined as rheumatoid arthritis, systemic lupus erythematosus, psoriasis, ankylosing spondylitis, systemic vasculitis, Crohn’s disease, and ulcerative colitis. ^e^ Defined as baseline hemoglobin < 13 g/dL for men and <12 g/dL for women. ABI, ankle-brachial index; CLTI, chronic limb-threatening ischemia; IC, intermittent claudication; PTA, percutaneous transluminal angioplasty.

## Data Availability

The data can be obtained from the corresponding author upon reasonable request.

## Supplementary Materials

The following supporting information can be downloaded at https://www.mdpi.com/article/10.3390/jcm13133991/s1. Table S1: Influence of lower limb revascularization on the parameters of macro- and microcirculation of the foot the total collective (*n* = 29).
