# Supplementary material for: The Effect of Revascularization on Lower Limb Circulation Parameters in Symptomatic Peripheral Arterial Disease
Source: J Clin Med. 2024 Jul 8;13(13):3991. doi: 10.3390/jcm13133991 (PMC11242648; doi:10.3390/jcm13133991)
Supplement: Supplementary file 1 [file jcm-13-03991-s001.zip › jcm-3051708-supplementary.pdf]

Table S1. Influence of lower limb revascularization on the parameters of macro- and microcirculation of the foot the total collective (n= 29).

Parameter, Position	PRE	PERI	POST	F/U
Flow (AU) supine	48.99 ± 37.86	90.85 ± 60.96[1]	79.12 ± 50.74[2]	69.96 ± 42.15[4]
Flow (AU) elevated	20.82 ± 14.63	56.06 ± 52.25[1]	45.85 ± 39.50[3]	51.56 ± 37.75[4]
SO2 (%) supine	54.75 ± 18,52	59.07 ± 18.93[1]	55.21 ± 16.75[2]	59.09 ± 13.45[4]
SO2 (%) elevated	35.10 ± 17.38	34.04 ± 19.78[2]	39.13 ± 19.22[3]	47.43 ± 22.12[4]
rHb (AU) supine	68.39 ± 19.08	74.45 ± 19.48[1]	69.72 ± 15.02[2]	77.69 ± 14.55[4]
rHb (AU) elevated	50.49 ±17.09	56.67 ± 18.66[1]	54.55 ± 16.84[3]	63.65 ± 15.67[4]

Data presented as mean ± standard deviation.
Flow (AU): relative microvascular blood flow (in arbitrary units);
rHb (AU): relative microvascular amount of hemoglobin (in arbitrary units);
SO2 (%): postcapillatory oxygen saturation of hemoglobin (percentage);
PRE = before; PERI = immediately after; POST = during the postoperative hospital stay, at the earliest one day after; F/U = 28-59 days after revascularization
[1]n=28 [2]n=27 [3]n=26 [4]n=18
